# Herbal-derived bioactive compounds in stroke recovery: from neuroprotective mechanisms to clinical evidence and translational challenges

**DOI:** 10.3389/fneur.2026.1902978

**Published:** 2026-10-16

**Authors:** Xi-zhe Sun, Jia-lu Lv, Lan-xin Gu, Ya-ge Li, Xiao-mao Li, Ye Li, Yu-meng Luo

**Affiliations:** 1 Drug Research and Development Technology Park, Hainan Medical University, Haikou, Hainan, China; 2 Key Laboratory of Basic and Application Research of Beiyao, Heilongjiang University of Chinese Medicine, Harbin, China; 3 The Second Affiliated Hospital of Heilongjiang University of Chinese Medicine, Harbin, Heilongjiang, China; 4 Traditional Chinese Medicine College, Bozhou University, Bozhou, China; 5 School of Pharmacy, Jiangsu Food and Pharmaceutical Science College, Huai'an, Jiangsu, China; 6 College of Pharmacy, Chongqing Three Gorges Medical College, Chongqing, China; 7 Traditional Chinese Medicine Biological Genetics, Heilongjiang Province Double First-Class Construction Interdiscipline, Harbin, China

**Keywords:** excitotoxicity, herbal-derived bioactive compounds, neuroinflammation, rehabilitation, stroke

## Abstract

Stroke is a leading cause of death and long-term disability worldwide, driven by pathophysiological processes including excitotoxicity, oxidative stress, neuroinflammation and apoptosis. Current pharmacological and non-pharmacological interventions primarily restore perfusion or support rehabilitation but do not fully address multifactorial secondary injury. Herbal-derived bioactive compounds possess antioxidant, anti-inflammatory and neuroprotective activities. Preclinical studies show that they limit neuronal injury and modulate excitotoxicity, oxidative stress, neuroinflammation, apoptosis, blood–brain barrier disruption and angiogenesis. Clinical trials suggest that these compounds can complement conventional treatment and improve functional or cognitive outcomes. This review synthesizes evidence on their mechanisms of action, therapeutic roles, combination strategies and research gaps. Herbal-derived bioactive compounds offer accessible adjuncts for stroke management, but their safe and effective integration into rehabilitation practice requires formulation standardization and large-scale clinical trials.

## 1 Introduction

A significant socioeconomic and healthcare burden Stroke is considered to be among the most common causes of mortality and long-term disability in the world. Ischemic stroke is characterized by the abrupt cessation of blood flow to the brain in ischemic stroke (IS) or breakage of a cerebral vessel in hemorrhagic stroke, which leads to the occurrence of a series of pathophysiological processes such as excitotoxicity, oxidative stress, neuroinflammation, and apoptotic cell death. Although thrombolytic therapy and mechanical thrombectomy have improved the management of acute stroke, the therapeutic window is still quite limited and most patient end up with neurological deficits, affecting the quality of life. There is, therefore, a dire need to find a solution to post-stroke rehabilitation and neuroprotection (1).

Over the past couple of years, there has been growing interest in exploring the therapeutic value of herbal-derived bioactive compounds. The flavonoids, alkaloids, terpenoids, and saponins, have been reported to possess neuroprotective ability in terms of antioxidant activity, anti-inflammatory activity, and anti-apoptotic activity. Studies in animal models of stroke have demonstrated in experimental research that they play a role in attenuating neuronal damage, increasing synaptic plasticity, and stimulating neurogenesis, and clinical studies indicate that they may be helpful in enhancing functional recovery when used in combination with rehabilitation programs (Figure 1). Notably, their multitarget activity provides a mechanistic rationale for evaluation as adjuncts to conventional care; comparative efficacy, safety, affordability and adherence remain insufficiently established (2–4).

**Figure 1:**
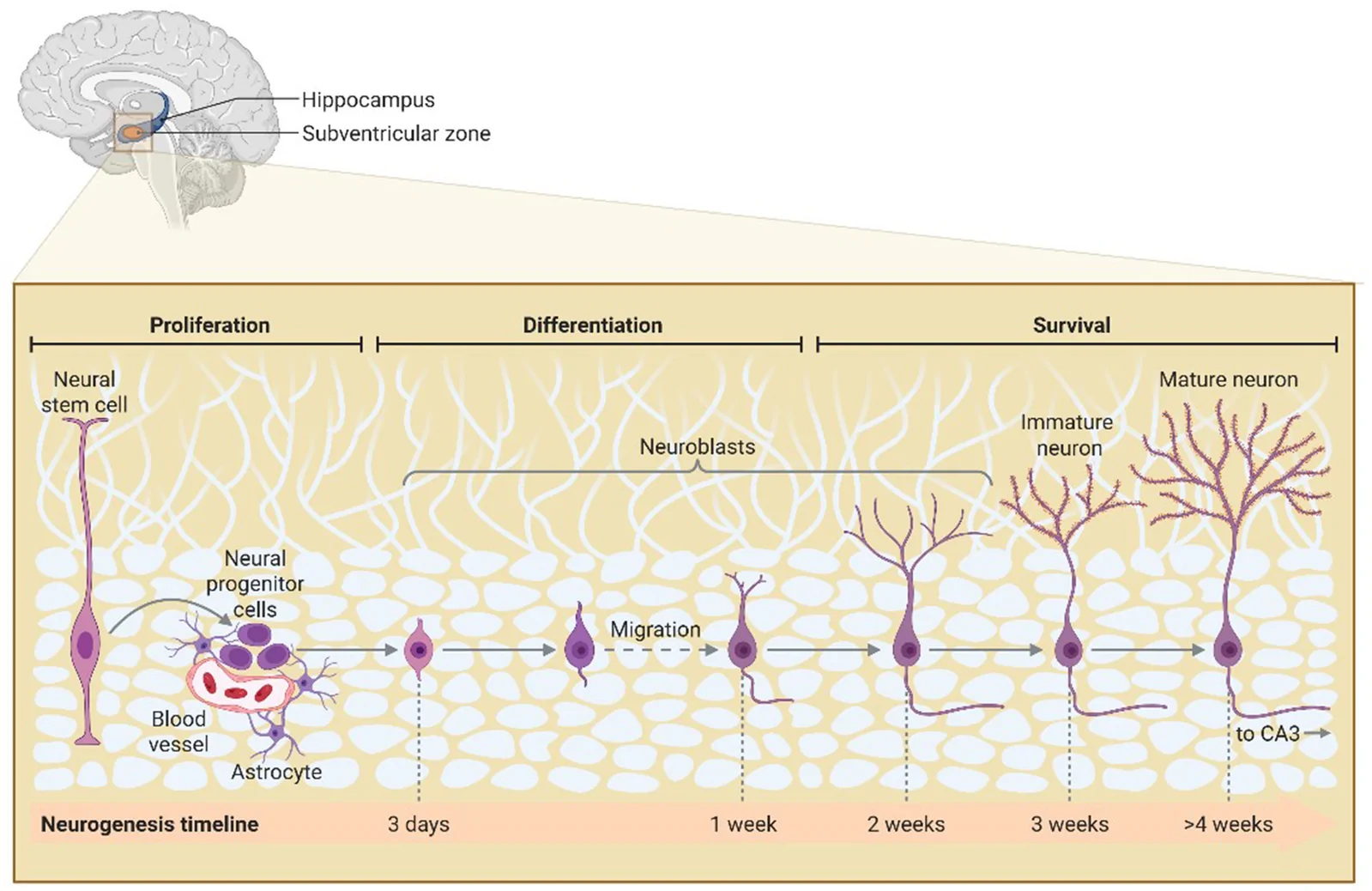
Schematic timeline of adult hippocampal neurogenesis. The process comprises three major stages: proliferation of neural stem cells within the hippocampal neurogenic niche to generate neural progenitor cells; differentiation and migration of neuroblasts; and progressive maturation and long-term survival of immature neurons. Over more than 4 weeks, newborn neurons develop mature neuronal features, establish synaptic connections, and integrate into hippocampal circuitry, with axonal projections extending toward the CA3 region (116).

To define the therapeutic value of these herbal-derived bioactive compounds for stroke recovery, it is necessary to conduct a comprehensive collation and integrative appraisal of existing preclinical and clinical evidence. This would allow not only a critical evaluation of their neuroprotective efficacy but also identify the current limitations, challenges in translation and future research directions. This review thus summarizes experimental and clinical evidence on bioactive compounds of herbal origin, and their mechanism of action and therapeutic effects in stroke recovery.

Relevant literature published up to September 2026 was identified through searches of PubMed, Web of Science, the China National Knowledge Infrastructure Database (CNKI) and Wanfang database. Search terms included combinations of “stroke,” “ischemic stroke,” “hemorrhagic stroke,” “stroke recovery,” “rehabilitation,” “herbal-derived bioactive compounds,” “polyphenols,” “terpenoids,” “oxidative stress,” “neuroinflammation,” and “clinical trial,” using Boolean operators where appropriate. English- and Chinese-language peer-reviewed publications were considered. Studies were selected according to their relevance to stroke pathophysiology, neuroprotective mechanisms, preclinical evidence, clinical outcomes, pharmacokinetic characteristics, safety, and translational challenges associated with herbal-derived bioactive compounds. Original experimental and clinical studies were prioritized, while high-quality reviews were used to provide broader mechanistic and clinical context. Duplicate records and publications that were clearly unrelated to the scope of this review were excluded.

## 2 Pathophysiology of stroke

### 2.1 Ischemic stroke

The most prevalent form is IS, which causes about 80–85 percent of all stroke cases around the world. It is caused by obstruction of brain arteries, most often by the presence of thrombus or embolism. IS may result in focal neurological deficits based on the location of the blockage and the duration of the blockage. The affected brain tissue may be subdivided into two larger areas, the ischemic core and the penumbra. Necrotic death of neurons occurs very quickly in the core region as a result of extreme energy loss. The hypoperfused penumbra still has some metabolic activity and may be salvaged (5, 6).

On a molecular scale, the IS causes a cascade of damaging processes within several minutes of its onset. The abrupt reduction in cerebral blood flow depletes cellular energy and triggers pathological responses, including excitotoxicity, neuroinflammation, oxidative stress and other related events. All of these effects increase cell death and secondary brain damage. These mechanisms are key to developing neuroprotective therapies, such as bioactive compounds of herbal origin (5, 7).

#### 2.1.1 Energy failure and excitotoxicity

After an IS, cerebral oxygen and glucose availability falls sharply, causing rapid neuronal adenosine triphosphate (ATP) depletion and energetic failure (8). ATP loss impairs Na^+^/K^+^-ATPase activity, promoting Na^+^ and Ca^2+^ influx, K^+^ efflux, cellular oedema and membrane depolarization. Membrane depolarization promotes excessive vesicular glutamate release from presynaptic terminals while impairing astrocytic glutamate reuptake (9). Excess glutamate overactivates N methyl D aspartate receptors (NMDARs), thereby causing a massive and sustained influx of Ca^2+^ into neurons, which induces intracellular calcium overload and subsequently leads to mitochondrial calcium overload and depolarization. In addition to NMDARs, glutamate activates calcium permeable alpha amino 3 hydroxy 5 methyl 4 isoxazolepropionic acid receptors (CP-AMPARs), promoting Na^+^ and Ca^2+^ influx, membrane depolarization and cellular swelling (10, 11).

Intracellular calcium overload activates calcium calmodulin dependent protein kinase II (CaMKII) and sustains the activation of Ca^2+^-dependent effectors, including phospholipases and proteases, thereby disrupting cellular architecture and intracellular homeostasis. Moreover, mitochondrial uptake of excess Ca^2+^ causes mitochondrial calcium overload and promotes the generation of reactive oxygen species (ROS). Accumulated ROS damages membrane structures and mitochondrial function, exacerbating glutamate excitotoxicity and calcium overload and thereby promoting secondary brain injury (Figure 2A) (12, 13).

**Figure 2:**
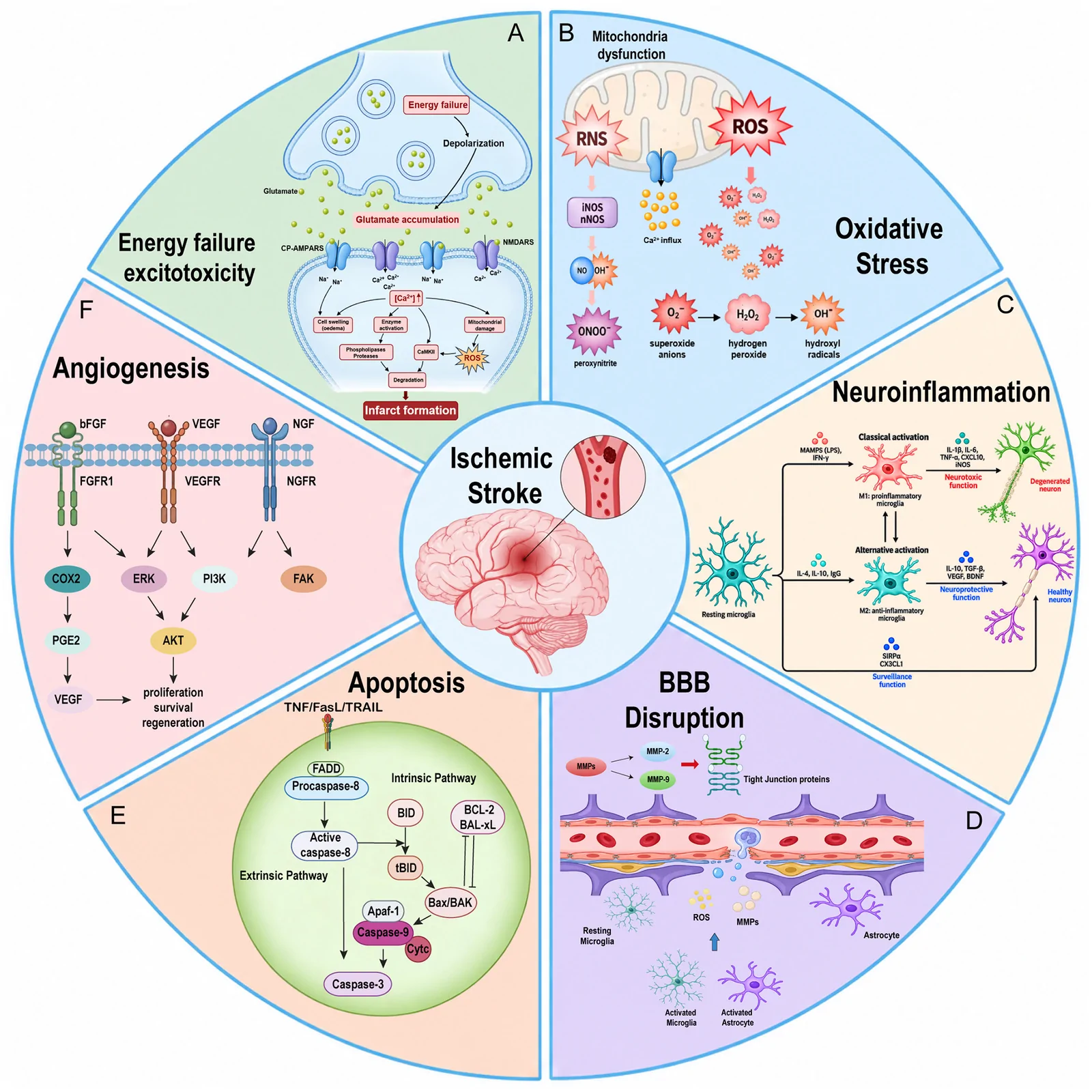
Pathophysiological mechanisms of IS. **(A)** Energy failure and excitotoxicity: ATP depletion leads to calcium overload and neuronal injury. **(B)** Oxidative stress: mitochondrial dysfunction promotes the accumulation of ROS and RNS. **(C)** Neuroinflammation: activated microglia release pro-inflammatory cytokines. **(D)** BBB disruption: ROS and MMPs degrade tight junction proteins. **(E)** Apoptosis: extrinsic and intrinsic pathways jointly lead to programmed neuronal death. **(F)** Angiogenesis: promotes endothelial-cell proliferation, migration and vascular remodeling.

#### 2.1.2 Oxidative stress

Oxidative stress is particularly prominent during the acute phase of IS, when hypoxia and reperfusion drive excessive production of ROS and reactive nitrogen species (RNS). Mitochondrial dysfunction is a major source of ROS, including superoxide anion (${\text{O}}_{2}^{-}$), hydroxyl radical (•OH) and hydrogen peroxide (H_2_O_2_) (14). Excess ROS reacts with membrane lipids, proteins and DNA, causing structural and functional damage. ROS further exacerbate mitochondrial dysfunction, activate inflammatory cascades and promote deleterious processes including excitotoxicity and apoptosis, thereby sustaining IS injury (15).

RNS are an important class of free radicals involved in poststroke brain injury, including NO and ONOO^−^. Following IS, calcium overload activates neuronal nitric oxide synthase (nNOS), while induction of inducible nitric oxide synthase (iNOS) further augments NO production. NO reacts with superoxide to generate ONOO^−^, triggering lipid peroxidation, mitochondrial dysfunction and apoptosis, thereby exacerbating injury induced by ischaemia and reperfusion. ROS and RNS also activate multiple cell death pathways. Apoptosis can further increase ROS and RNS production, establishing a feed-forward cycle that intensifies injury in IS (Figure 2B) (16, 17).

#### 2.1.3 Neuroinflammation

After an IS, dying cells activate pattern recognition receptors (PRRs) on brain cells, inducing adhesion molecule expression and proinflammatory cytokine release that initiate neuroinflammation (13). Microglia are the principal immune cells in neuroinflammation after stroke, particularly within the ischaemic penumbra. During IS, interleukin 4 (IL-4) drives an M2 microglial phenotype characterized by the release of immunoregulatory mediators [e.g., interleukin 10 (IL-10), transforming growth factor beta (TGF-β), vascular endothelial growth factor (VEGF), brain derived neurotrophic factor (BDNF)] and neuroprotective activity. Microglia can also acquire a proinflammatory M1 phenotype, leading to the release of proinflammatory cytokines [e.g., interleukin 1 beta (IL-1β), tumor necrosis factor alpha (TNF-α), and interleukin 18 (IL-18)] (18). Resting microglia mediate constant immune surveillance and sense microenvironmental cues to preserve central nervous system homeostasis (Figure 2C) (19).

#### 2.1.4 BBB disruption

The blood brain barrier (BBB) is a specialized component of the neurovascular unit, comprising endothelial cells, pericytes, astrocytes, microglia and the basement membrane. Its restrictive architecture and selective transport systems prevent blood cells, pathogens and toxic substances from entering the central nervous system while regulating nutrient exchange, thereby preserving microenvironmental homeostasis. Tight junction proteins between endothelial cells form the structural core of the BBB and restrict the passive diffusion of water-soluble solutes (20, 21).

BBB injury after IS is progressive and multiphasic. During the acute phase, ROS and matrix metalloproteinase 2 (MMP-2) disrupt tight junction proteins, increasing BBB permeability and permitting immune-cell infiltration that amplifies neuroinflammation. Subsequently, matrix metalloproteinase 9 (MMP-9) released by astrocytes and microglia further degrades the basement membrane and tight junction proteins, leading to BBB dysfunction and vasogenic oedema. During the subacute phase, approximately 1–3 weeks after onset, barrier repair is initiated, with angiogenesis as a major component. BBB dysfunction also contributes to the narrow therapeutic window for thrombolytic treatment (Figure 2D) (22–24).

#### 2.1.5 Apoptosis

Following IS, neurons in the ischaemic penumbra undergo programmed apoptosis through the extrinsic (death receptor) pathway and intrinsic (mitochondrial) pathway. This process constitutes an important mechanism for the removal of irreversibly damaged cells. After an IS, activated immune cells in the inflammatory microenvironment secrete a panel of pro-apoptotic ligands, including TNF-α, Fas ligand (FasL) and tumor necrosis factor related apoptosis inducing ligand (TRAIL). These ligands bind to their cognate death receptors on the plasma membrane, thereby initiating the extrinsic apoptotic pathway. Receptor activation recruits Fas associated death domain protein (FADD), which promotes procaspase-8 activation to generate active caspase-8. Caspase-8 can directly activate caspase-3, causing nuclear DNA fragmentation and apoptosis, or cleave Bid to generate tBid, thereby linking extrinsic signals to the intrinsic apoptotic pathway (25, 26).

IS promotes intracellular calcium overload, which activates calpains and facilitates Bid cleavage to tBid. tBid antagonizes the anti-apoptotic activities of B cell lymphoma 2 (Bcl-2) and B cell lymphoma extra large (Bcl-xL) while promoting the conformational activation and outer mitochondrial membrane localization of BCL2 associated X apoptosis regulator (Bax) and BCL2 antagonist killer 1 (Bak). This sequence increases outer mitochondrial membrane permeabilisation and releases cytochrome c and other apoptogenic factors. Cytochrome c subsequently associates with apoptotic protease activating factor 1 (Apaf-1) and procaspase-9 to form the apoptosome, which activates caspase-9 and downstream caspase-3, ultimately executing apoptosis (Figure 2E) (16, 25, 27).

#### 2.1.6 Angiogenesis

Studies suggest that programmes of angiogenesis are initiated in the ischaemic penumbra 3–4 days after IS. Vascular remodeling, sprouting angiogenesis and the formation of mature arteries from existing arterioles improve cerebral perfusion and metabolism, thereby promoting neurological recovery (28).

Endothelial cells at the infarct border enter a proliferative state as early as 12–24 h after onset. Hypoxia upregulates VEGF expression, and VEGF together with NO mediates local vasodilation and promotes plasma protein extravasation. Reactive astrocytes remodel the extracellular matrix to provide a scaffold for endothelial migration. Upon binding to its endothelial receptors, VEGF activates extracellular signal regulated kinase (ERK) and phosphoinositide 3 kinase (PI3K)/protein kinase B (AKT) signaling (Supplementary Figure S2), thereby driving endothelial proliferation and migration and promoting sprouting angiogenesis (28). In pericytes, fibroblast growth factor receptor 1 (FGFR1) can activate AKT/ERK signaling, increase basic fibroblast growth factor (bFGF) expression and upregulate prostaglandin E2 and VEGF, thereby supporting neurological recovery. Nerve growth factor (NGF) also exerts effects that promote angiogenesis through focal adhesion kinase (FAK) and PI3K/AKT signaling. Therapeutically enhancing angiogenesis in the penumbra may reduce infarct volume, limit neuronal apoptosis and help reconstruct the neurovascular network, representing a potential strategy for IS (Figure 2F) (29, 30).

Overall, IS involves multiple interconnected pathological mechanisms and unfolds through a dynamic interplay between injury and repair, which together shape disease progression and clinical outcomes.

### 2.2 Hemorrhagic stroke

Hemorrhagic stroke is the leading cause of all stroke and is more likely to be fatal compared to ischemic stroke, as illustrated in Supplementary Table S1. It is a condition in which a weakened cerebral blood vessel bursts causing bleeding in or surrounding the brain tissue. Intracerebral hemorrhage (ICH) and subarachnoid hemorrhage (SAH) are the most common ones. The key contributing factors are hypertension, rupture of an aneurysm, and arteriovenous malformations. The accumulated blood suddenly creates a mechanical pressure, and the neurons are damaged very quickly (31).

Pathophysiology of hemorrhagic stroke is not limited by the initial rupture. The development of hematoma causes compression of brain structures, increase of intracranial pressure and decrease of cerebral perfusion. Oxidative stress and inflammatory reactions are caused by the breakdown products of blood such as hemoglobin and iron. These secondary mechanisms play a role in edema, neuronal apoptosis, and additional tissue destruction. The complications of this type deteriorate clinical prognosis and frequently restrict treatment effectiveness (32). Therefore, antioxidant and anti-inflammatory compounds obtained using herbs have potential clinical applications in rehabilitation (33).

## 3 Current therapeutic strategies and limitations

### 3.1 Pharmacological interventions

The management of acute stroke is still dominated by pharmacological treatment, with most of the focus being on restoring cerebral perfusion. Thrombolytic agents such as recombinant tissue plasminogen activator (rt-PA) are also emerging in ischemic stroke to break the clot and re-establish blood circulation (34). The therapeutic window, however, is small and falls within a 4.5 h range of the symptom onset. Mechanical thrombectomy has increased treatment opportunities but is restricted to a few cases. Delayed presentation to hospital renders many patients ineligible for these interventions (35).

In the case of hemorrhagic stroke, pharmacological treatment is primarily supportive and not curative. To suppress hypertension and decrease the chances of hematoma growth, antihypertensive medications are usually used. There are rare situations in which hemostatic agents can be applied to restrict active bleeding. Antiplatelet and anticoagulant drugs exert preventive effects in both types of stroke but carry a risk of haemorrhagic complications (36).

Current pharmacological approaches primarily address acute events and insufficiently target secondary injury, including oxidative stress, inflammation and neuronal apoptosis. Neuroprotective candidates have shown limited clinical translation, while safety concerns and adverse effects restrict their use (16).

### 3.2 Non-pharmacological approaches

Non-pharmacological therapeutic methods play a crucial role in the treatment of stroke, especially long-term rehabilitation and functional recovery. Some non-pharmacological therapeutic methods are illustrated in Supplementary Figure S1. Motor exercises and task-specific training are central components of motor recovery. Occupational therapy enhances patients' capacity to perform activities of daily living. Communication problems and post-stroke aphasia require speech and language therapy. Collectively, these treatments are the basis of multidisciplinary rehabilitation of stroke (37).

There are also other innovative methods gradually being applied to rehabilitation therapy. In robot-assisted training and exercises based on virtual reality, intensive and task-oriented training enhances motor learning. Transcranial magnetic stimulation (TMS) and transcranial direct current stimulation (tDCS) represent some of the non-invasive forms of brain stimulation that have potential to facilitate neuroplasticity (38). These new techniques are in addition to conventional rehabilitation and facilitate neuronal functional recovery (39).

Another very important aspect of non-pharmacological management is lifestyle changes. A routine aerobic workout, healthy diet, and quitting smoking can reduce the risk of recurrent stroke. Psychosocial support and cognitive training help alleviate post-stroke depression in patients. Non-pharmacological therapies, when combined with pharmacological treatments and novel natural interventions, may offer a more holistic approach to stroke management (40).

### 3.3 Challenges in stroke rehabilitation

Barriers to stroke rehabilitation commonly exist on both the systemic and patient levels. Limited access to rehabilitation services, high costs and delayed treatment initiation compromise recovery. Depression, cognitive impairment, and deficits can easily prevent engagement with long-term rehabilitation programs. Older patients and those with comorbidities may experience delayed functional recovery. These are some of the factors that lead to chronic disability and low quality of life (41). On the clinical front, the existing treatments have failed to fully meet the challenge of multifactoriality of neuronal injury. The absence of multitargeted, combined measures is restrictive in the overall performance of the currently used protocols. These challenges support the evaluation of complementary interventions, including herbal-derived bioactive compounds, in adequately designed clinical studies (42).

## 4 Herbal-derived bioactive compounds: an overview

### 4.1 Definition and classification

Herbal-derived bioactive compounds are naturally present chemical substances produced by extraction of medicinal plants that can have a positive impact on human health. These compounds tend to target multiple pathways at the same time (43).

All these compounds are in broad categories that are recognized according to their chemical structure and biological activity. Polyphenols, such as flavonoids and phenolic acids, mainly exert antioxidant and anti-inflammatory effects. The flavonoid quercetin can alleviate oxidative stress and enhance synaptic plasticity. Alkaloids, such as berberine, modulate the neurotransmitter system and regulate cerebral blood circulation. Terpenoids and saponins have also been demonstrated to exert neuroprotective and vasoactive effects. For example, ginsenosides promote neurogenesis and vascular repair, and modulate ion channels and neurotransmitter release. The framework of classification offers a basis through which they can systematically review their experimental and clinical findings in stroke rehabilitation (44, 45).

### 4.2 Therapeutic features and potential advantages

Herbal-derived bioactive compounds serve as a unique adjunct to conventional stroke therapy. Their multitargeted mode of action confers therapeutic value during both the acute and recovery phases of stroke. Curcumin activates nuclear factor erythroid 2 related factor 2 (Nrf2) mediated antioxidant pathways and inhibits nuclear factor kappa B (NF-κB) signaling, thereby attenuating oxidative injury and inflammatory responses (46, 47). It also activates the Wnt/β-catenin signaling pathway to promote neural cell proliferation, as well as the differentiation and maturation of newborn neurons (48). This expanded therapeutic range is well-suited to pathologically complex diseases such as stroke.

The other major benefit is that they have a relatively good safety profile. A clinical trial of Panax notoginseng saponins (PNS) showed that 3 months of treatment significantly increased the rate of functional independence relative to the control group, while the incidence of serious adverse events was comparable between groups (49). These findings support the clinical efficacy and safety of PNS as an adjunctive treatment for IS.

Accessibility and cost-effectiveness also contribute to the arguments that support the role of herbal bioactive compounds in the treatment of stroke. In contrast to advanced pharmacological or surgical treatment, most of these compounds can be obtained in common plants. This may be particularly instrumental in resource-sensitive settings, where only restriction is the access to special therapies (50).

## 5 Neuroprotective mechanisms of herbal-derived bioactive compounds in preclinical studies

Herbal-derived bioactive compounds are increasingly being explored in stroke research, because their capacity to modulate multiple pathophysiological processes offers a distinct mechanistic rationale. This Review synthesizes available experimental evidence indicating that these compounds exert neuroprotective effects throughout the entire course of stroke (Table 1).

**Table 1:** Summary of mechanisms of herbal-derived bioactives in stroke treatment.

Components	Animal/cells	*In vivo*/*In vitro*	Type	Targets and signal pathways	Effects	References
Curcumin	Mice, MCAO/R HBMECs, OGD/R	*In vivo* *In vitro*	IS	↑ADRB2, Nrf2, HO-1, SOD, GSH ↓DNMT1, MDA, IL-1β, IL-6, TNF-α Inhibit JAK2/STAT3 pathway Activate ADRB2/Nrf2/HO-1 pathway	↓Infarct volume and neuronal damage ↓Inflammation and oxidative injury ↑Vascular repair and BBB integrity	(51)
Resveratrol	Rats, MCAO/R	*In vivo*	IS	↑Nrf2, NQO1, GSH, SOD T-AOC ↓ASC, caspase-1, iNOS, IL-1β, TNF-α Inhibit TLR4/NF-κB/AIM2 pathway Activate Nrf2/NQO1 pathway	↓Infarct volume, and neuronal damage ↓Inflammation and oxidative injury ↓Pyroptosis	(52)
	Rats, MCAO/R PC12 cells, OGD/R	*In vivo* *In vitro*	IS	↑Bcl-2, p62; ↓TNF-α, IL-6, IL-1β, Bax, caspase-3, Beclin-1, LC3-II/I	↓Inflammation, apoptosis ↓Excessive autophagy	(53)
Gomisin N	Mice, MCAO/R PC12 cells, OGD/R	*In vivo* *In vitro*	IS	↑p62; ↓LC3-II/I Activate PI3K/AKT/mTOR pathway	↓Infarct area and excessive autophagy ↑Neurological and locomotor function	(117)
Verbascoside	Mice, MCAO/R SK-N-SH cells, OGD/R	*In vivo* *In vitro*	IS	Inhibit LCN2/ALOX5 pathway Activate SLC7A11/GPX4 pathway	↓Infarct area and neuronal damage ↓Ferroptosis and apoptosis	(118)
Wedelolactone	Mice, MCAO/R PC12 cells, OGD/R	*In vivo* *In vitro*	IS	↑GSH; ↓Fe^2^^+^, LDH, MDA, ROS Activate HIF-1α/SLC7A11/GPX4 pathway	↓Infarct area and neuronal damage ↓Ferroptosis and oxidative injury	(119)
Nodakenin	Rats, I/R PC12 cells, OGD/R	*In vivo* *In vitro*	IS	↑SOD, GPX4, FTH1 ↓TNF-α, IL-1β, IL-6, MDA, Fe^2^^+^ Inhibit NF-κB pathway Activate PI3K/AKT pathway	↓Infarct area and histological injury ↓Ferroptosis, inflammation and oxidative injury	(120)
Piceatannol	Mice, MCAO/R Neuronal cells, OGD/R	*In vivo* *In vitro*	IS	↓ACSL4, FPN1, ROS, MDA Inhibit USP14/GPX4 pathway	↓Pyroptosis and inflammation ↓Lipid peroxidation and ferroptosis	(121)
Danshensu sodium	Mice, tMCAO Neuronal cells, OGD/R	*In vivo* *In vitro*	IS	↓RAGE, IL-1β, IL-6, TNF-α, IL-18 Inhibit NLRP3/GSDMD/ASC/caspase-1 pathway	↓Pyroptosis and inflammation	(122)
Salvianolic acid A	Rats, electrocoagulation HBMECs, H_2_O_2_	*In vivo* *In vitro*	IS	↑VEGF-A, Ang-1, ZO-1, occluding, claudin-5; ↓Fe^2^^+^, LDH, MDA, ROS Activate AKT/Nrf2 pathway	↑Angiogenesis and vascular maturation ↑BBB integrity ↓Oxidative stress	(123)
Salvianolic acid B	Mice MCAO Brain microvascular endothelial cells, OGD/R	*In vivo* *In vitro*	IS	↑HO-1, GPX4; ↓ACSL4, TFR1 Activate Nrf2/Keap1 pathway	↓Infarct volume and neuronal damage ↑BBB integrity ↓Ferroptosis	(124)
Quercetin	Mice MCAO SH-SY5Y, OGD/R	*In vivo* *In vitro*	IS	↑Bcl-2, SOD, HO-1; ↓Bax, ROS, MDA Activate Nrf2/STAT3/SLC6A3 pathway	↓Infarct volume and neuronal damage ↓Oxidative stress and apoptosis	(54)
Baicalin	Mice, tMCAO Astrocyte	*In vivo* *In vitro*	IS	↓GFAP, IL-6, IL-1β, MCP-1, TNF-α	↑Neurological outcomes ↓Inflammation, astrocyte activation	(125)
	Mice, endovascular puncture HT22 cells, SAH model	*In vivo* *In vitro*	SAH	↓NLRP3 Activate PI3K/AKT/GPX4 pathway	↓Ferroptosis and neuronal damage	(126)
EGCG	Rats, MCAO/R BV2 microglia, OGD/R	*In vivo* *In vitro*	IS	↑IL-4, IL-10 ↓IL-1β, TNF-α, CD32, iNOS Activate Nrf2/HO-1 pathway	↓Infarct area and hippocampal injury ↓Inflammation, excitotoxicity and ferroptosis	(127)
	Rats, autologous arterial blood SK-N-SH cells, 10 μM of hemoglobin	*In vivo* *In vitro*	ICH	↑Nrf2, HO-1, FTH1, GPX4 ↓ROS, MDA Activate Nrf2/Keap1 pathway	↓Haematoma volume and tissue injury ↑Neurobehavioural outcomes ↓Ferroptosis	(128)
Puerarin	Rats, MCAO HT22 cells, OGD/R	*In vivo* *In vitro*	IS	↑SLC7A11, GPX4; ↓ACSL4 Inhibit caspase-1/GSDMD Activate SLC7A11/GPX4 pathway	↓Ferroptosis and pyroptosis	(129)
	Rats, collagenase IV	*In vivo*	ICH	Activate cGAS-STING pathway	↑BBB integrity, remyelination ↓White-matter injury, apoptosis	(130)
Apigenin	Rats, MCAO	*In vivo*	IS	Activate AMPK pathway	↓Oxidative stress and inflammation	(131)
	Mice spontaneous ICH	*In vivo*	ICH	Activate PRDX2/Trx1 pathway	↓ICH incidence, hemorrhage volume	(132)
Hydroxysafflor yellow A	Rats, tMCAO/R Neural stem cells	*In vivo* *In vitro*	IS	Inhibit MAPK/ERK pathway	↑Apoptosis ↑Neuronal differentiation	(133)
	Rats, autologous-blood Hemin-stimulated BV2 cells	*In vivo* *In vitro*	ICH	↓TNF-α, IL-1β, IL-6, HMGB1, Microglial activation Inhibit RIPK1/RIPK3/MLKL pathway	↓Oedema, neuroinflammation ↓Inflammatory, necroptosis and apoptosis	(134)
Tetrahydropiperine	Rats, MCAO PC12 cells, OGD	*In vivo* *In vitro*	IS	↑p62, LC3-I ↓Atg7 and Beclin-1 Activate PI3K/AKT/mTOR pathway	↓Infarct area and neuronal injury ↓Excessive autophagy	(58)
Voacangine	HT22 cells, OGD/R	*In vitro*	IS	↓ROS; ↑SOD Activate PI3K/AKT/FoxO pathway	↓Excitotoxicity, oxidative stress and ferroptosis	(59)
Berberine	Rats CIRI	*In vivo*	IS	↓TNF-α, IL-1β, GRP78, p-PERK, p-eIF2α, QRICH1	↓Infarct area and neural deficits ↓ERS and inflammatory	(135)
Berberine	Transgenic zebrafish, atorvastatin induced ICH	*In vivo*	ICH	↓VE-cad, Occludin	↓Inflammation and locomotor injury ↑Integrity of vascular walls	(136)
Berberine	Intravascular puncture HT22 cells, oxygenated hemoglobin injury	*In vivo* *In vitro*	SAH	↓GSK3β, IL-1β Inhibit caspase-1/GSDMD pathway	↓Oedema and BBB permeability ↓Neuronal pyroptosis and inflammation.	(137)
Tetramethylpyrazine	Mice, MCAO Astrocytes, OGD	*In vivo* *In vitro*	IS	↑p-AKT/AKT, ZO-1, SOD, GSH ↓ROS and MDA	↓Infarct area and oxidative stress ↑BBB integrity	(138)
Tetramethylpyrazine	Mice, collagenase VII	*In vivo*	ICH	↑p62, GPX4, FSP1 ↓MDA, ROS, NCOA4 Activate PI3K/AKT pathway	↑Neurological function ↓Hemorrhage volume, ferroptosis	(139)
Matrine	Mice, MCAO	*In vivo*	IS	Modulate UCP2/AMPK pathway	↑Neurological function ↓Oxidative stress and apoptosis	(140)
Matrine	Mice, autologous blood injection	*In vivo*	ICH	↓PKC, PLC, Fe^2^^+^, ACSL4, Bax, IL-6, IL-1β, TNF-α; ↑GPX4, Bcl-2, ZO-1 Inhibit HMGB1/RAGE mediated ERK/NF-κB pathway	↓Hematoma volume, BBB leakage ↓Inflammation, apoptosis, ferroptosis	(141)
Oxymatrine	Rats, MCAO BV-2 microglia, OGD	*In vivo* *In vitro*	IS	↓ROS, Fe^2^^+^, MDA, DRP1 ↑HO-1, SLC7A11, FTH, GPX4, OPA1 Activate Keap1/Nrf2 pathway	↑Neurological outcomes ↓Ferroptosis and lipid peroxidation ↑Mitochondrial homeostasis	(142)
Piperine	Rats, MCAO BV-2 microglia, OGD	*In vivo* *In vitro*	IS	↓NLRP3, IL-1β, ASC, IL-18, Inhibit caspase-1/GSDMD-N pathway	↑Neurological and motor function ↓Oedema, pyroptosis, inflammation	(143)
Palmatine	Rats, MCAO BV2 microglia	*In vivo* *In vitro*	IS	Activate PI3K/AKT/mTOR/HIF-1α pathway	↑Sphingolipid homeostasis ↓Microglial activation	(144)
Catalpol	Neural stem cells	*In vitro*	IS	Activate SDF-1α/CXCR4 pathway	↑Neurogenesis and angiogenesis	(145)
Geniposide	Mice, MCAO/R Neuronal OGD/R	*In vivo* *In vitro*	IS	Inhibit cGAS/STING pathway	↓Neuronal injury, excessive autophagy	(146)
Geniposide	Mice, collagenase IV	*In vivo*	ICH	↓IL-1β, TNF-α, ROS Inhibit NF-κB pathway	↓Oedema, inflammation and oxidative stress	(147)
L-Borneolum	Rats, MCAO/R hCMEC/D3 BBB co-culture, OGD	*In vivo* *In vitro*	IS	Modulate MFSD2A/Cav-1 pathway	↑BBB transcellular transport, cerebral blood flow and neurological outcome	(148)
Ginkgolide B	Rats, tMcao BV2 microglia, OGD/R	*In vivo* *In vitro*	IS	↓TRIM45, NF-κB, IL-1β, IL-6, TNF-α	↓Oedema, apoptosis and BBB injury	(149)
Triptolide	Mice, MCAO/R	*In vivo*	IS	Modulate Fractalkine/CX3CR1 pathway	↓Neuronal injury, microglial activation	(150)
Asiatic acid	Rats, SAH	*In vivo*	SAH	↓ACOX1	↓Neuronal injury, ferroptosis	(151)
Astragaloside IV	Rats, MCAO/R SH-SY5Y cells, OGD/R	*In vivo* *In vitro*	IS	Activate p62/Keap1/Nrf2 pathway	↓Sensorimotor dysfunction ↓Ferroptosis	(61)
Ginsenoside Rb1	Mice, I/R model PC12 cells, OGD/R	*In vivo* *In vitro*	IS	↑Bcl-2 interaction Inhibit ATM/CHK2/Beclin-1 pathway	↓Excessive autophagy and oxidative stress	(62)
Ginsenoside Rg1	Mice, MCAO SK-N-AS and SH-SY5Y cells, OGD/R	*In vivo* *In vitro*	IS	↑p-P70S6K, p-RPS6 Activate AKT/mTOR/ULK1 pathway	↓Excessive autophagy, apoptosis and lysosomal activity	(152)
Glycyrrhizic acid	Rats, MCAO Hippocampal neurons, OGD/R	*In vivo* *In vitro*	IS	Activate PI3K/AKT pathway	↓Infarct volume, apoptosis, inflammation	(153)
Diammonium glycyrrhizinate	Mice, collagenase	*In vivo*	ICH	↓VCAM-1, IL-1β, IL-6, TNF-α, MMP-9 Activate PI3K/AKT pathway	↓BBB leakage, inflammation, oedema ↓Oxidative stress and apoptosis	(154)
Gastrodin	Rats, MCAO/R PC12 cells, OGD/R	*In vivo* *In vitro*	IS	↑GPX4; ↓ACSL4, LPCAT3	↓Ferroptosis and lipid peroxidation	(63)
Chrysophanol	OGD/R models	*In vitro*	IS	Inhibit ZNF460/TRAF6 pathway	↓Necroptosis	(64)
Tanshinone IIA	Rast, MCAO/R PC12 cells, OGD/R	*In vivo* *In vitro*	IS	↓caspase-1 cleavage, IL-1β, IL-18 Activate KLF4/CHIP/NLRP3 pathway	↑Neurological function ↓Pyroptosis	(155)
	Mice, collagenase LPS stimulated glial cells	*In vivo* *In vitro*	ICH	↓PAD4, H3Cit Inhibit NLRP3/caspase-1 pathway	↑Sensorimotor impairment ↓NETosis and inflammation	(156)
	Rats, SAH	*In vivo*	SAH	↓IL-6, MCP-1, TNF-α, IL-1β, IL-18 Inhibit NF-κB/NLRP3 pathway	↓Inflammation	(157)
Emodin	Rats, MCAO Astrocyte neuron, OGD/R	*In vivo* *In vitro*	IS	↓IL-1β, TNF-α, IFN-γ, EMP1, GFAP Modulate 11β-HSD1/AKT pathway Inhibit NF-κB pathway	↑A1 to A2 astrocyte shift	(158)
Shikonin	Primary astrocytes, OGD/R	*In vitro*	IS	Inhibit ERK1/2-SP1-SLC7A10 pathway	↑A1 to A2 astrocyte shift	(159)
Geraniin	Rats, MCAO/R PC12 cells, OGD/R	*In vivo* *In vitro*	IS	↓LDH, MDA, NO, nNOS, Bax, caspase-3 Activate Nrf2/HO-1 pathway	↓Oxidative stress, excitotoxicity and apoptosis	(65)

“↑”, upregulated; “↓”, downregulated.

### 5.1 Phenylpropanoids

Phenylpropanoids, which are present in fruits, vegetables, tea and medicinal plants, scavenge reactive oxygen species and alleviate oxidative stress. Experimental evidence indicates that curcumin, reverses hypermethylation of the β_2_-adrenergic receptor (ADRB2), thereby suppressing the Janus kinase 2/signal transducer and activator of transcription 3 (JAK2/STAT3) signaling pathway while activating the Nrf2/ heme oxygenase 1 (HO-1) antioxidant pathway. These effects attenuate inflammation and oxidative damage, and promote blood–brain barrier repair and angiogenesis (51). Resveratrol (Res) is a bioactive compound extracted from the rhizomes of *Polygonum cuspidatum* Sieb. et Zucc. In rat middle cerebral artery occlusion reperfusion (MCAO/R) models, Res reduces infarct volume and alleviates neuronal damage. By targeting the Toll like receptor 4 (TLR4)/NF-κB/ absent in melanoma 2 (AIM2) and Nrf2/ NAD (P) H:quinone oxidoreductase 1 (NQO1) signaling axes, it exerts anti-neuroinflammatory and antioxidative stress effects, which ameliorate neurological deficits and emotional-cognitive dysfunction (52). In addition, resveratrol suppresses nuclear receptor subfamily 3 group C member 2 (NR3C2) expression and directly modulates tripartite motif containing 28 (TRIM28) transcription in an NR3C2-dependent manner, thus alleviating ischemia-induced apoptosis and autophagy dysregulation and conferring neuroprotection (53).

### 5.2 Flavonoid

Flavonoids possess diverse biological activities, including antimicrobial, antioxidant, and anti-inflammatory effects. Through reactions catalyzed by polyphenol oxidases and peroxidases, flavonoids can scavenge ROS and other free radicals, thereby limiting radical generation. Quercetin, a representative flavonoid, reduces the expression or release of TNF-α, IL-1β and IL-6. In an oxygen glucose deprivation reoxygenation (OGD/R) model of brain microvascular endothelial cells (BMECs), quercetin has also been associated with enhanced Nrf2-dependent antioxidant responses, reduced endoplasmic reticulum stress, and increased expression of the tight junction proteins claudin-5 and zonula occludens 1 (ZO-1) (54). Baicalin, a flavonoid isolated from the dried roots of *Scutellaria baicalensis*, exhibits antioxidant, anti-apoptotic and anti-inflammatory activities. Experimental studies suggest that baicalin improves neurological deficits, restores mitochondrial membrane potential, suppresses apoptosis and enhances mitophagy. It downregulates NLR family pyrin domain containing 3 (NLRP3) inflammasome activation and inhibits TLR4/ myeloid differentiation primary response 88 (MyD88)/NF-κB signaling, thereby limiting caspase-1-mediated (55). Baicalin also activates the Nrf2/antioxidant response element (ARE) pathway, increases the expression of HO-1, SOD and glutathione peroxidase (GSH-Px), scavenges ROS and suppresses lipid peroxidation (56).

### 5.3 Alkaloids

Alkaloids are a group of heterogeneous nitrogenous substances, which are present in most medicinal plants and which are associated with substantial pharmacological actions. The compounds mimic neurotransmitter systems, enhance cerebral blood flow, and decrease excitotoxicity by controlling glutamate release (57). Tetrahydropiperine (THP) is a natural arylpentanamide alkaloid isolated from black pepper (*Piper nigrum* L.). In a permanent middle cerebral artery occlusion (pMCAO) rat model, THP improved neurological function, reduced infarct volume, and preserved neurons in the ischaemic penumbra. These effects were associated with activation of the PI3K/Akt/ mechanistic target of rapamycin (mTOR) signaling pathway and reduced expression of the autophagy related 7 (Atg7) and Beclin-1. THP may therefore attenuate ischaemic brain injury by limiting excessive autophagy and sustaining neuronal survival (58). Voacangine is a natural monoterpenoid indole alkaloid with emerging neuroprotective potential. In OGD/R injured mouse hippocampal neuronal cell line (HT22) hippocampal neuronal cells, voacangine reduced cytotoxicity and reactive oxygen species accumulation while increasing superoxide dismutase activity. It also activated the PI3K/Akt/ forkhead box O (FoxO) signaling pathway, thereby limiting oxidative stress and ferroptosis. These findings suggest that voacangine may preserve hippocampal neuronal survival during ischaemia reperfusion injury (59).

### 5.4 Terpenoids and saponins

Terpenoids and saponins are isoprenoid-derived natural products with complex, polycyclic hydrophobic scaffolds that confer diverse biological activities. Accumulating evidence suggests that these compounds exert multitarget neuroprotective effects in ischaemic stroke (60). Astragaloside IV, a saponin isolated from *Astragalus membranaceus* var. *mongholicus*, may inhibit ferroptosis by upregulating the activating transcription factor 3 (ATF3)/ fat mass and obesity associated protein (FTO) axis to reduce acyl CoA synthetase long chain family member 4 (ACSL4) methylation. It also regulates the sequestosome 1 (p62)/ Kelch like ECH associated protein 1 (Keap1)/Nrf2 signaling axis, thereby reducing malondialdehyde levels and preserving glutathione availability (61). Ginsenoside Rb1 (GRb1) is a major tetracyclic triterpenoid saponin in ginseng. In MCAO/R mice, GRb1 alleviated neurological deficits and histopathological damage. These effects were associated with reduced oxidative stress and restraint of excessive autophagy. GRb1 decreased the phosphorylation of ataxia telangiectasia mutated (ATM) and checkpoint kinase 2 (CHK2), while enhancing the interaction between Beclin-1 and Bcl-2, consistent with inhibition of autophagy initiation. In OGD/R injured PC12 cells, GRb1 similarly reduced cell injury and intracellular reactive oxygen species accumulation. ATM may therefore represent a candidate direct target through which GRb1 coordinates redox homeostasis and autophagy (62). A portion of terpenoids also regulate MAPK signaling pathways that control cell survival and regeneration (Figure 3). It has been shown in pre-clinical trials that terpenoids can help improve cognitive functioning and motor recovery, which is why they make good candidates to be included in stroke rehabilitation programs (60).

**Figure 3:**
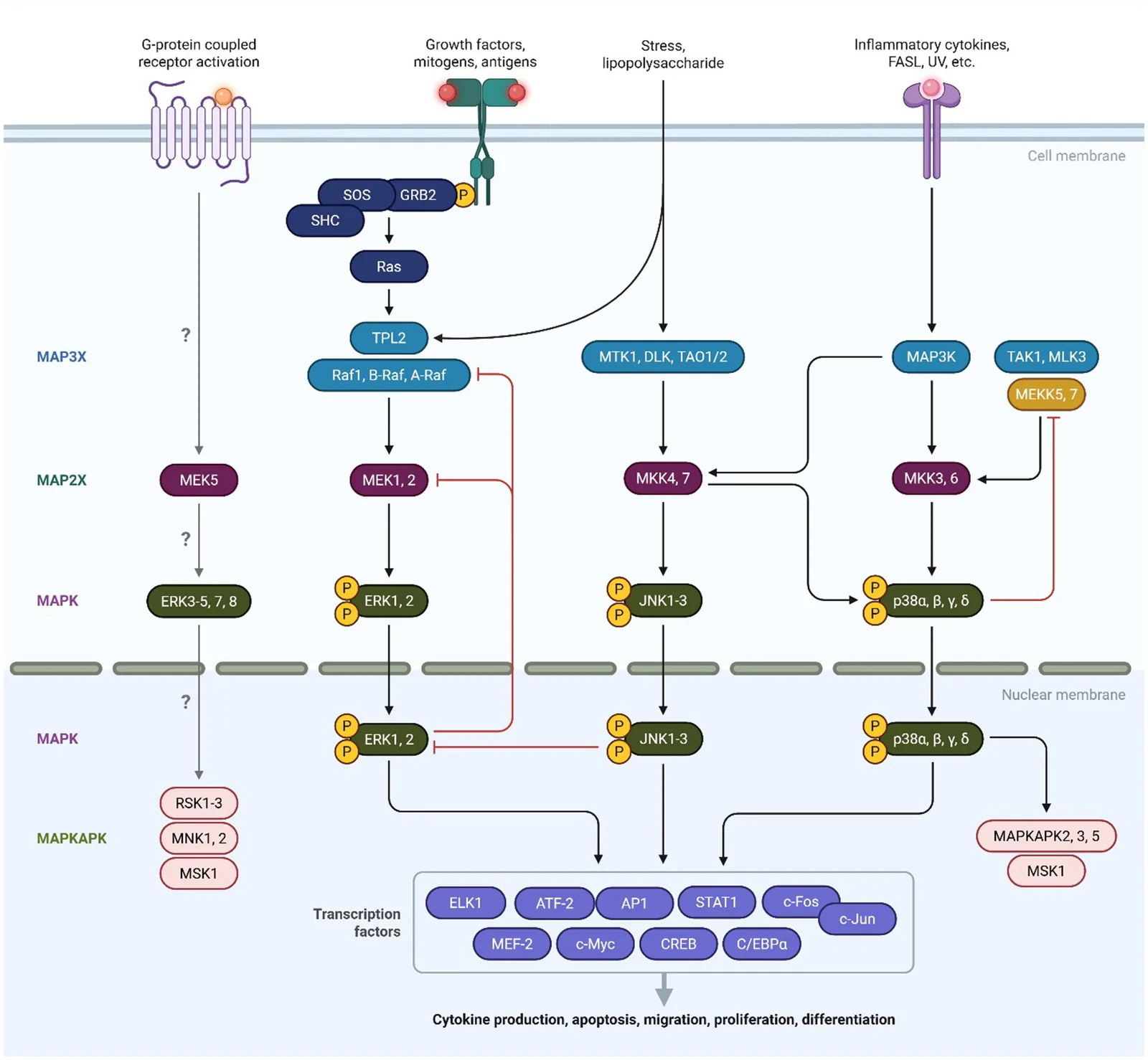
Regulation of cell death and survival by the MAPK signaling pathway. Extracellular stimuli activate the MAPK cascade through sequential phosphorylation. Phosphorylated signals are transmitted to the nucleus, where they regulate downstream transcription factors and ultimately determine biological processes including cytokine production, apoptosis, proliferation, migration and differentiation. Solid arrows indicate activation or phosphorylation, whereas red lines indicate inhibitory regulation.

### 5.5 Other phytochemicals

Other natural products, including glycosides, quinones and tannins, display pharmacological activities that may attenuate oxidative injury, promote neuroprotection and suppress ferroptosis (57, 60). Gastrodin is a phenolic glycoside isolated from the dried rhizome of *Gastrodia elata*, an orchidaceous medicinal plant. It inhibits ferroptosis by regulating the cystine/glutamate antiporter (xCT)/glutathione peroxidase 4 (GPX4) and acyl-CoA synthetase long-chain family member 4 (ACSL4)/lysophosphatidylcholine acyltransferase 3 (LPCAT3) pathways. These actions decrease malondialdehyde levels while increasing superoxide dismutase activity and glutathione availability (63). Chrysophanol (CHR) is a naturally occurring anthraquinone in *Rheum* species. By suppressing the zinc finger protein 460/tumor necrosis factor receptor-associated factor 6 (ZNF460/TRAF6) axis, CHR limits post-stroke necroptosis and attenuates neuronal injury (64). Geraniin is a tannin isolated from *Phyllanthus amarus*. It activates the nuclear factor erythroid 2-related factor 2/heme oxygenase 1 (Nrf2/HO-1) pathway, reduces lactate dehydrogenase activity and decreases malondialdehyde, nitric oxide and neuronal nitric oxide synthase levels, thereby attenuating oxidative injury and neuronal apoptosis (65). Overall, these phytochemicals represent promising preclinical candidates for multitarget stroke therapy.

## 6 Clinical studies on herbal bioactive compounds in stroke rehabilitation

### 6.1 Randomized controlled trials

Randomized controlled trials (RCTs) offer the most evidence when analyzing the therapeutic potential of bioactive compounds of herbal origin in the rehabilitation of stroke patients. Numerous RCTs have examined the impact of herbal extracts and preparations of herbal-derived bioactive compounds on functional outcomes in patients with stroke. During early recovery from IS, Ginkgo biloba extract EGb 761^®^ was administered as an adjunct to standard care. A prospective, randomized, multicentre, open-label trial enrolled 201 patients with mild to moderate IS. The intervention group received oral EGb 761^®^ plus standard care for 24 weeks. At week 24, EGb 761^®^ produced a significantly greater improvement in Montreal Cognitive Assessment (MoCA) scores than standard care alone, with parallel benefits in verbal learning and Shape Trail Test (66). Additionally, DL-3-n-butylphthalide (NBP), a phthalide derived from Apium graveolens, has been evaluated as an adjunct to reperfusion therapy for acute ischaemic stroke (AIS). A multicentre, double blind, placebo-controlled RCT enrolled 1,216 patients receiving intravenous rt-PA or endovascular treatment including mechanical thrombectomy. NBP was initiated within 6 h of symptom onset, administered intravenously for the first 14 days, and subsequently given as oral soft capsules. At day 90, 56.7% of patients receiving NBP achieved a favorable modified Rankin Scale (mRS) outcome, compared with 44.0% in the placebo group, while serious adverse event rates were comparable (67). Findings have shown that the use of these compounds as supplements to conventional therapy improves cognitive and general quality of life. These results indicate that herbal bioactives can be used to supplement standard rehabilitation regimens.

Despite these encouraging findings, the current RCT evidence has several methodological limitations that affect its robustness and generalizability. Some trials used open-label or non-placebo-controlled designs, whereas others were limited by single-center recruitment, small sample sizes, short treatment or follow-up periods, or enrollment of specific stroke subtypes and restricted patient populations. In addition, all newly included RCTs were conducted in China, which may limit the geographic generalizability of the findings. Considerable heterogeneity in stroke stage, intervention formulations, dosing regimens, routes of administration, comparators, and outcome measures further complicates direct comparisons across trials. These limitations reduce confidence in the durability and broader applicability of the reported benefits. Future studies should therefore employ adequately powered, multicenter, rigorously controlled designs with standardized interventions and outcome measures, longer follow-up, and more geographically diverse populations to strengthen the evidence base for incorporating herbal-derived bioactive compounds into evidence-based stroke rehabilitation (49, 68). The characteristics of the 10 included RCTs are summarized in Table 2.

**Table 2:** The clinical RCTs involved in stroke.

Type	Characteristics	Inclusion criteria	Intervention group	Control group	Primary outcome	Adverse events	Study limitations	References
Multicenter, double-blind, RCT	China; IS; IG, *n* = 1,487; PG, *n* = 1,479; 3 months	Age 18–75 years; IS diagnosed within 14 days of onset; prestroke mRS ≤ 1; NIHSS 4–15	Xuesaitong soft capsule: 60 mg/capsule; orally twice daily	Placebo soft capsule	mRS ≤ 2: 89.3 vs. 82.4%; OR = 1.95, 95% CI 1.56–2.44; *P* < 0.001	SAE: 1.0 vs. 1.1%; *P* = 0.85	Broad enrollment window and short follow-up; limited stage-specific and long-term assessment	(49)
Multicenter, open-label, RCT	China; AIS; IG, *n* = 100; CG, *n* = 101; 24 weeks	Age ≥50 years; AIS confirmed by MRA; diagnosis within 7–14 days of onset; NIHSS ≤ 20	*Ginkgo biloba* extract EGb 761^®^ tablet: 40 mg/tablet; orally three times daily	Standard care	MoCA improvement: 2.92 vs. 1.33 points; 95% CI 0.51–2.67; *P* < 0.001	AE: 11.1 vs. 9.9%	Open-label, non-placebo design; increased bias risk and reduced outcome reliability	(66)
Multicenter, double-blind, RCT	China; AIS; IG, *n* = 607; PG, *n* = 609; 90 days	Age ≥18 years; AIS diagnosed within 6 h of onset; NIHSS 4–25	NBP injection: 100 mL/dose; intravenously twice daily; NBP soft capsule: 0.2 g/capsule; orally three times daily	Placebo injection and placebo soft capsule	mRS ≤ 2: 56.7 vs. 44.0%; OR = 1.70, 95% CI 1.35–2.14; *P* < 0.001	AE: 23.1 vs. 24.5%; *P* = 0.65	Short follow-up; limited assessment of sustained efficacy and long-term safety	(67)
Multicenter, double-blind, RCT	China; AIS; GDLM group, *n* = 1,725; PG, *n* = 1,723; 14 days	Age 18–80 years; AIS diagnosed within 14 days of onset; prestroke mRS ≤ 1; NIHSS 4–24	GDLM: 5 m/dose; intravenously once daily	Placebo injection	mRS ≤ 1 at 14 days: 50.8 vs. 44.1%; OR = 1.31, 95% CI 1.15–1.50; *P* < 0.001	AE: 17.6 vs. 17.3%; *P* = 0.83	Short treatment and follow-up; limited assessment of durable recovery and long-term safety	(160)
Single-center, open-label, RCT	China; cardioembolic stroke; IG, *n* = 58; CG, *n* = 58; 14 days	Age ≥18 years; cardioembolic stroke confirmed by CTA/MRA; puncture within 24 h of onset; prestroke mRS ≤ 1; NIHSS >6	DGMI: 25 mg/dose, 25 mg intravenously once daily	Standard care	Median NIHSS 7.0 vs. 10.5; Median mRS 2.5 vs. 3.5; OR = 3.490, 95% CI 1.077–11.316; *P* = 0.037	ICH: 1 vs. 0 case	Single-center, open-label, small sample; increased bias risk and limited generalizability	(161)
Multicenter, double-blind, RCT	China; AIS; IG, *n* = 333; PG, *n* = 113; 10 days	Age 40–80 years; AIS diagnosed within 72 h of onset; prestroke mRS ≤ 1; baseline mRS >2; NIHSS 4–17	Shuxuening injection: 5 mL/dose; 20 mL intravenously once daily	Placebo injection	mRS ≤ 2: 82.98 vs. 65.18%; between-group difference 95% CI 8.09%−27.51%; *P* < 0.0001	AE: 49.85 vs. 56.90%; *P* = 0.1898	Short treatment and follow-up; limited assessment of sustained functional recovery	(162)
Multicenter, double-blind, RCT	China; AIS; GTC group, *n* = 100; EGB group, *n* = 101; PG: *n* = 101; 12 weeks	Age 18–75 years; ACIS with essential hypertension; prestroke mRS ≤ 1; stroke onset >2 and < 12 weeks; NIHSS 6–22	GTC group: 250 mg Getongtongluo capsule + 40 mg EGB placebo; orally twice daily	EGB group: EGB + GTC placebo; Placebo group: GTC placebo + EGB placebo	BI relative independence rate ≥75% at 12 weeks: both GTC and EGB groups higher than placebo; *P* < 0.05	AE: GTC 10.55%, EGB 8.33%, placebo 11.67%	Modest sample and restricted hypertensive population; limited generalizability	(163)
Single-center, double-blind, RCT	China; lacunar stroke; IG, *n* = 40; PG, *n* = 31; 12 months	Age ≥18 years; clinically diagnosed lacunar stroke	*Salicornia ramosissima* extract capsule: 0.5 g/capsule; 1 g orally daily	Placebo capsule	Cognitive outcomes favored intervention; 95% CI 0.08–4.36; *P* = 0.042	AE: 26 vs. 43%; *P* = 0.234	Single-center, small lacunar-stroke sample; limited statistical power and generalizability	(164)
Multicenter, double-blind, RCT	China; AIS; IG, *n* = 1,002; PG, *n* = 1,005; 90 days	Age 35–75 years; IS confirmed by CT/MRI; diagnosis within 72 h of onset; prestroke mRS ≤ 1; NIHSS 4–22	Tongxinluo capsule: 4 capsules per dose; orally three times daily	Placebo capsule	mRS 0–1: 65.8 vs. 59.1%; OR = 1.33, 95% CI 1.11–1.60; *P* = 0.002	AE: 38.6 vs. 38.4%; *P* = 0.95	Acute-stage recruitment and short follow-up; limited later-stage and long-term assessment	(107)
Multicenter, double-blind, RCT	China; AIS; HSYAI, LG: *n* = 66; MG, *n* = 67; HG, *n* = 65; DZXXI group, *n* = 68; 14 days	Age 35–75 years; IS with BSS meeting CM diagnostic criteria; diagnosis within 7 days of onset; NIHSS 7–21	(1) LG: 25 mg/day, (2) MG: 50 mg/day, (3) HG: 70 mg/day; Intravenously once daily	DZXXI group: 30 mL/day	mRS ≤ 1: LG 33.33%, MG 56.72%, HG 58.46%, DZXXI 38.24%	AE: LG 51.43%, MG 50.00%, HG 62.32%, DZXXI 56.34%	Small dose groups and short follow-up; limited precision and long-term efficacy assessment	(165)

IG, intervention group; PG, placebo group; CG, control group; LG, low group; MG, medium group; HG, high group; GDLM, Ginkgolide meglumine injection; DGMI, Ginkgolide meglumine injection; HSYAI, hydroxysafflor yellow A injection; DZXXI, Dengzhan Xixin injection; AE, adverse events; SAE, serious adverse event.

### 6.2 Observational and cohort studies

The observational and cohort studies are critical real-life evidence on the use of herb-extracted bioactive compounds in the prevention and rehabilitation of stroke. According to hospital researches, Ginkgo biloba extract and ginseng preparation have been shown to improve cognitive abilities, fatigue, and activities of daily living among patients after a stroke. These results imply practical, rehabilitative use value (69, 70).

Observational studies do have limitations because of confounding factors such as lifestyle, concomitant medication and dietary differences. This also reduces the accuracy and makes causality difficult due to the poor patient compliance and self-reported intake. In spite of these shortcomings, this kind of study offers useful evidence on long-term safety, feasibility, and patient satisfaction that would otherwise be underrepresented in randomized controlled trials (71, 72).

### 6.3 Combination with non-pharmacological interventions

Combining bioactive compounds of herbal origin with Non-Pharmacological Interventions used in stroke rehabilitation is a new direction in improving treatment outcomes. In contrast to being used independently, when used together with physical, occupational and speech therapy these compounds enhance neural responsiveness to training. Peng et al. conducted a retrospective comparative study of Ginkgo biloba extract combined with low-frequency repetitive transcranial magnetic stimulation (LF-rTMS) in 93 patients with ischaemic stroke. After 4 weeks, the clinical response rate based on the National Institutes of Health Stroke Scale (NIHSS) was 96.77% in the combination treatment group, exceeding that in the LF-rTMS group (80.65%) and the regular treatment group (54.84%). The combined therapy also increased SOD levels, reduced malondialdehyde (MDA) and endothelin-1 concentrations, and improved indices of cerebral oxygen metabolism. Moreover, the 6-month recurrence rate was 3.23% in the combination treatment group, lower than in the LF-rTMS group (19.35%) and the regular treatment group (25.81%) (73). Such a combination treatment is indicative of a more comprehensive recovery model than a strictly pharmacological treatment.

## 7 Limitations and knowledge gaps

### 7.1 Bioavailability

Herbal-derived bioactive compounds offer new opportunities for stroke therapy; however, limited bioavailability remains a major barrier to their clinical translation. Ginsenosides provide a representative example. The oral bioavailability of dammarane-type saponins such as Rb1, Rb2, and Rg1 is generally low, with the absolute bioavailability of Rb1 in rats reported to be only 4.35%. Their dammarane skeleton and multiple glycosyl substituents reduce lipophilicity and membrane permeability, while P-glycoprotein (P-gp)-mediated efflux can further return absorbed ginsenosides to the intestinal lumen and reduce net absorption (74, 75). Polyphenol-rich herbal constituents face different but related pharmacokinetic constraints. Many occur as glycosides that require deglycosylation before absorption, after which the resulting aglycones may undergo extensive glucuronidation, sulfation, and methylation in the intestinal wall and liver, resulting in low circulating concentrations of unchanged parent compounds (76).

These general pharmacokinetic limitations are manifested differently among individual bioactive compounds. Curcumin has poor aqueous solubility and limited membrane permeability and is also subject to rapid elimination and systemic clearance, collectively resulting in very low oral exposure (77). Quercetin is absorbed mainly in the intestine but subsequently undergoes extensive phase II metabolism in the intestinal wall and liver, generating glucuronidated, sulfated, and methylated conjugates. The intestinal microbiota can further convert quercetin and its glycosides into smaller phenolic metabolites, substantially altering the chemical forms present in the circulation after oral administration (78). Thus, the major pharmacokinetic constraints involve different processes, including solubility, intestinal permeability, metabolic transformation, efflux, and clearance. These compound differences among herbal-derived bioactive compounds are highly relevant to clinical translation. Effect concentrations identified in preclinical studies may not be achievable *in vivo*, and circulating metabolites may differ substantially from the parent compounds evaluated *in vitro*. Therefore, dose selection, exposure–response assessment, and formulation development should be guided by the predominant pharmacokinetic limitation of each compound rather than by a uniform strategy.

### 7.2 Herb–drug interactions

With the growing use of herbal medicines as complementary and alternative therapies, unexpected interactions and adverse reactions arising from concomitant medication have also increased, raising concern regarding herb–drug interactions (HDIs). HDIs arise predominantly through pharmacokinetic mechanisms (79, 80). Numerous herbal constituents induce or inhibit the expression or activity of cytochrome P450 (CYP450) enzymes and P-glycoprotein (P-gp). Studies have shown that co-administration of curcumin and atorvastatin does not enhance lipid-lowering efficacy but increases atorvastatin plasma exposure, thereby increasing the risk of liver injury. This interaction may be associated with curcumin-mediated inhibition of P-gp and cytochrome P450 family 3 subfamily A member 2 (CYP3A2) activity in rats (81, 82). HDIs can also arise through pharmacodynamic synergy or antagonism. For example, co-administration of *Ginkgo biloba* extract with clopidogrel or other antiplatelet agents may further suppress arachidonic acid pathway mediated platelet aggregation and increase the risk of bleeding events. Thus, HDIs represent a double-edged sword, and appropriate guidance for their use is essential to ensure medication safety (83, 84).

### 7.3 Safety and heterogeneity

Many herbal-derived bioactive compounds have long histories of dietary or medicinal use. However, adverse effects and toxicity vary according to compound identity, formulation, dose, exposure duration and individual characteristics (85, 86). However, some bioactive compounds can elicit cytotoxic responses, resulting in neurotoxicity, hepatotoxicity and nephrotoxicity. Prolonged administration of high-dose berberine may cause diarrhea, vomiting, haemorrhagic intestinal inflammation and even death (87, 88). In addition, high doses of polyphenols may exert hepatic or renal toxicity. For example, apigenin at 100 mg/kg may cause hepatotoxicity, whereas higher doses may induce sedation and muscle relaxation. The toxic effects of these compounds are determined by multiple factors, including dose, exposure duration and individual characteristics (89–91).

Chemical heterogeneity and limited standardization are also important determinants of safety and reproducibility. Soil conditions, climate, medicinal plant parts, processing methods and extraction procedures can substantially alter both the qualitative and quantitative profiles of bioactive constituents (92). Inadequate standardization of raw materials, extraction procedures, marker compounds and batch-level quality control further reduces comparability across preparations and may compromise reproducibility (93). *Rhodiola* harvested from different geographical regions shows marked differences in the composition and abundance of its pharmacologically active components (94). At the formulation level, the bioavailability of different commercial preparations derived from the same plant extract may differ by several fold. A comparison of four products containing *Withania somnifera* (L.) Dunal found that the AUC of total withanolides differed by as much as 267-fold across products (95). Such heterogeneity also contributes to inconsistent findings in HDI studies, in which the same herb may induce drug metabolism or transport in one setting but inhibit it in another. Ultimately, this heterogeneity and limited standardization make it difficult to establish reproducible product specifications and clinically reliable dosing for herbal bioactive compounds.

### 7.4 Challenges in clinical translation

Clinical research on herbal-derived bioactive compounds currently faces methodological bottlenecks that require urgent resolution. Beyond small sample sizes and short follow-up periods, methodological limitations of herbal-medicine RCTs include inadequate allocation concealment, incomplete blinding, unclear specification of primary outcomes, insufficient sample-size estimation, suboptimal handling of missing data and limited use of intention-to-treat analyses (96, 97). These limitations hinder accurate assessment of the true efficacy and tolerability of bioactive compounds and preclude the identification of delayed organ injury, leaving the long-term safety of chronic administration unresolved. In addition, negative or null findings are difficult to retrieve, resulting in underrepresentation of negative evidence in systematic reviews (98, 99). Selective outcome reporting may further distort the evidence base by preferentially presenting statistically significant outcomes, time points or analyses. In AIS RCTs, outcome reporting bias has been associated with overestimation of treatment benefits and underestimation of adverse effects (100). Differences between experimental stroke models and human disease limit the translational relevance of preclinical findings. Preclinical studies often use young, healthy animals under highly controlled conditions, whereas stroke patients are typically older, clinically heterogeneous, and frequently have comorbidities. These discrepancies in species, age, comorbidities, disease models, treatment timing, and outcome measures can limit the direct extrapolation of efficacy observed in animals to clinical benefit in patients (101, 102). Together, low bioavailability, toxicity and formulation heterogeneity, methodological limitations of RCTs, publication and selective-reporting bias, and the preclinical–clinical translational gap increase uncertainty in the evidence base and impede the development of clinical guidelines and evidence-based standards (103).

## 8 Conclusion and future perspectives

Herbal-derived bioactive compounds show bright prospects in the field of stroke rehabilitation development due to their antioxidant, anti-inflammatory, and neuroprotective activity. Both experimental and clinical evidence has shown that they have the potential to improve functional recovery. The significant advantage of these compounds is that their multitargeted mechanisms overcome the multifaceted pathophysiology of stroke. They coordinate a complex treatment protocol via regulation of neuronal plasticity, vascular healing, and metabolism. This multidimensional profile makes herbal bioactives complements to other current rehabilitation approaches. Although encouraging, a lot of the data are still in preliminary stages. There is a reason to be cautious yet optimistic (104).

Although preclinical studies have demonstrated the therapeutic potential of herbal-derived bioactive compounds for stroke, their translation into clinical practice remains challenging. First, low bioavailability and complex pharmacokinetic properties, together with compositional heterogeneity arising from differences in raw-material sources and extraction methods, represent major barriers to consistent therapeutic effects. Future research should prioritize the establishment of quality-control and standardization systems that implement quality management throughout the process from raw materials to finished products and provide full traceability. Advanced analytical approaches, including high-resolution mass spectrometry combined with chromatographic fingerprinting, should be used to achieve accurate quantification of herbal constituents and comprehensive quality assessment. At the pharmacokinetic level, the absorption, metabolism, target-organ distribution and exposure–response relationships of active constituents and their metabolites should be systematically characterized, together with standardized HDI studies. Dose optimization should therefore be guided by compound-specific pharmacokinetics, integrating dose gradients with measurements of systemic and brain exposure and pharmacodynamic biomarkers to define clinically relevant exposure–response relationships. For compounds limited by poor solubility, rapid metabolism, or inadequate brain delivery, formulation design should specifically target the predominant pharmacokinetic bottleneck. Beyond formulation-based approaches, structural modification may provide a complementary strategy for compounds with intrinsically unfavorable physicochemical properties. For example, curcumin derivatives, prodrugs, and molecular conjugates have been investigated to improve solubility, permeability, metabolic stability, and systemic exposure (77, 105). However, such modifications require further confirmation that the desired neuroprotective activity, brain disposition, and safety profile are retained.

In addition, clinical studies of herbal-derived bioactive compounds generally lack large, multicentre randomized controlled trials that meet international standards. Limited sample sizes, inconsistent trial protocols and insufficient systematic evaluation of long-term toxicity risks further hinder the generation of high-quality evidence-based medical evidence. To prove effectiveness and safety, large and standardized clinical trials should be conducted with a long-term follow-up study in the future. The other significant aspect here, is the formulating of quality-controlled formulations and dose-optimization plans to overcome the variability in plant extracts. The mechanisms can be better understood and the clinical applications can be refined using modern tools, including network pharmacology, multi-omics, and high-tech imaging. Morever, rational multi-component combinations may represent a potential strategy for addressing the multifactorial pathophysiology of stroke. Studies have shown that the combination of salvianolic acid B and ginsenoside Rg1 exerts greater protective effects than either component alone (106). In clinical practice, multi-component formulations such as Tongxinluo have also been increasingly investigated as therapeutic approaches for stroke recovery (107). Future studies should further integrate component-level standardization, pharmacokinetic interaction analyses, and formal assessments of synergistic effects to establish more rational combination strategies. These measures will make the research results more applicable to practice (Supplementary Figure S3) (108).

For compounds whose translation is constrained by poor solubility, rapid clearance, or limited brain delivery, advanced drug-delivery systems provide a complementary strategy to structural modification and dose optimization. Liposomes are classical delivery vehicles for natural products and can enhance drug stability while reducing toxicity (109). One study used scutellarin to neutralize the positive charge of berberine and developed a novel ionizable lipid for brain-targeted delivery. The resulting carrier retained dopamine receptor D3 (DRD3) binding affinity and BBB penetration capacity (110). Curcumin-loaded gelatin nanoparticles have also been developed for ischemic stroke and were shown in preclinical models to facilitate BBB delivery and attenuate oxidative stress and neuroinflammation (111). Nanoparticles can be generated by encapsulating or dissolving active pharmaceutical ingredients within polymeric matrices and are characterized by small particle size, strong targeting capacity and sustained drug release. Nanoformulations can substantially improve bioavailability and drug-loading efficiency, enhance BBB permeability and increase drug accumulation in the brain (112). In one study, quercetin-based polydopamine nanoparticles (Que@DAR NPs) were developed for ischaemic stroke. The nanoparticles were generated through oxidative self-assembly of dopamine around quercetin to facilitate brain-targeted delivery, enhance quercetin dispersion stability and enable responsive degradation in reactive oxygen species rich microenvironments. Que@DAR NPs also attenuated neuroinflammation and neuronal apoptosis, thereby enhancing neuroprotection (113). Despite these advances, the long-term *in vivo* metabolic stability and potential toxicity of nanomedicines remain key concerns. Moreover, the stringent quality-control requirements of delivery systems result in prolonged development cycles and high production costs, thereby hindering clinical translation.

In the context of rapid advances in artificial intelligence (AI), AI-driven formulation design has achieved over 99% accuracy in predicting nanoparticle performance, substantially improving drug-delivery efficiency and stability (114). Machine-learning models can directly predict the drug-loading efficiency and gene-delivery capacity of liposomes and polymeric nanoparticles from formulation composition (115). Future work should use AI trained on experimental data to predict optimal combinations of ionizable lipids and polymeric carriers, thereby addressing the low bioavailability and insufficient brain-targeting efficiency of herbal-derived bioactive compounds. Together with standardized quality-evaluation systems, these approaches may advance herbal-derived bioactive compounds toward precision-medicine applications.

## Glossary

ACIS, acute cerebral ischaemic stroke; ACOX1, acyl-CoA oxidase 1; ACSL4, acyl-CoA synthetase long-chain family member 4; ADRB2, β-2 adrenergic receptor; AE, adverse event; AIM2, absent in melanoma 2; AIS, acute ischaemic stroke; AKT, protein kinase B; ALOX5, arachidonate 5-lipoxygenase; Ang-1, angiopoietin-1; AP1, activator protein 1; Apaf-1, apoptotic protease-activating factor 1; ARE, antioxidant response element; ASC, apoptosis-associated speck-like protein containing a caspase recruitment domain; ATF-2, activating transcription factor 2; Atg7, autophagy-related 7; Bax, BCL2-associated X apoptosis regulator; BBB, blood-brain barrier; BBS, blood stasis syndrome; Bcl-2, B-cell lymphoma 2; Bcl-xL, B-cell lymphoma-extra large; BI, Barthel Index; BMECs, brain microvascular endothelial cells; Cav-1, caveolin-1; CD32, cluster of differentiation 32; C/EBPα, CCAAT/enhancer-binding protein alpha; c-Fos, cellular FBJ murine osteosarcoma viral oncogene homolog; cGAS, cyclic GMP-AMP synthase; CHIP, carboxyl terminus of HSC70-interacting protein; CHK2, checkpoint kinase 2; CI, confidence interval; CIRI, cerebral ischaemia-reperfusion injury; CM, Chinese medicine; c-Myc, cellular myelocytomatosis viral oncogene homolog; CREB, cAMP response element binding protein; CTA, computed tomography angiography; CX3CR1, C-X3-C motif chemokine receptor 1; DGMI, Diterpene Ginkgolides meglumine injection; DLK, dual leucine zipper kinase; DNMT1, DNA methyltransferase 1; DRP1, dynamin-related protein 1; DZXXI, Dengzhan Xixin injection; EGB, Ginkgo biloba extract; EGCG, epigallocatechin-3-gallate; eIF2α, eukaryotic initiation factor 2 α; ELK1, ETS-like gene 1; EMP1, epithelial membrane protein 1; ERK, extracellular signal-regulated kinase; ERS, endoplasmic reticulum stress; FAK, focal adhesion kinase; FGFR1, fibroblast growth factor receptor 1; FPN1, ferroportin 1; FSP1, ferroptosis suppressor protein 1; GDLM, Ginkgo Diterpene lactone meglumine; GFAP, glial fibrillary acidic protein; GPX4, glutathione peroxidase 4; GRB2, growth factor receptor-bound protein 2; GRP78, glucose-regulated protein 78; GSDMD-N, N-terminal gasdermin D; GSH, glutathione; GSK3β, glycogen synthase kinase 3 β; GTC, Getong Tongluo capsule; H3Cit, citrullinated histone H3; HIF-1α, hypoxia-inducible factor 1 α; HMGB1, high mobility group box 1; HO-1, heme oxygenase 1; HSYAI, hydroxysafflor yellow A injection; ICH, intracerebral hemorrhage; IFN-γ, interferon γ; IL-1β, interleukin-1 β; IL-4, interleukin-4; IL-6, interleukin-6; IL-10, interleukin-10; IL-18, interleukin-18; iNOS, inducible nitric oxide synthase; I/R, ischaemia/reperfusion; IS, ischaemic stroke; JAK2, Janus kinase 2; JNK, c-Jun N-terminal kinase; Keap1, kelch-like ECH-associated protein 1; KLF4, Krueppel-like factor 4; LC3-I, microtubule-associated protein 1 light chain 3-I; LCN2, lipocalin 2; LDH, lactate dehydrogenase; LPCAT3, lysophosphatidylcholine acyltransferase 3; LPS, lipopolysaccharide; MAP3K, mitogen-activated protein kinase kinase kinase; MAPKAPK, mitogen-activated protein kinase-activated protein kinase; MCAO/R, middle cerebral artery occlusion/reperfusion; MCP-1, monocyte chemoattractant protein 1; MDA, malondialdehyde; MEF-2, myocyte enhancer factor-2; MEK, mitogen-activated protein kinase kinase; MFSD2A, major facilitator superfamily domain-containing protein 2A; MLK3, mixed lineage kinase 3; MMP-9, matrix metalloproteinase 9; MNK, mitogen-activated protein kinase-interacting kinase; MoCA, Montreal Cognitive Assessment; MRA, magnetic resonance angiography; MRI, magnetic resonance imaging; mRS, modified Rankin Scale; MSK, mitogen- and stress-activated protein kinase; MTK1, MAP three kinase 1; mTOR, mechanistic target of rapamycin; NBP, DL-3-n-butylphthalide; NCOA4, nuclear receptor coactivator 4; NETosis, neutrophil extracellular trap formation; NF-κB, nuclear factor κB; NGF, nerve growth factor; NIHSS, National Institutes of Health Stroke Scale; NLRP3, NLR family pyrin domain-containing 3; nNOS, neuronal nitric oxide synthase; NQO1, NAD (P) H:quinone oxidoreductase 1; Nrf2, nuclear factor erythroid 2-related factor 2; OGD/R, oxygen-glucose deprivation/reoxygenation; OPA1, optic atrophy 1; OR, odds ratio; p38, p38 mitogen-activated protein kinases; p62, sequestosome 1; p70S6K, p70 ribosomal S6 kinase; PAD4, peptidyl arginine deiminase 4; PERK, protein kinase R-like endoplasmic reticulum kinase; PGE2, prostaglandin E2; PI3K, phosphoinositide 3-kinase; PKC, protein kinase C; PLC, phospholipase C; PRDX2, peroxiredoxin 2; QRICH1, glutamine-rich protein 1; Raf, rapidly accelerated fibrosarcoma kinase; RAGE, receptor for advanced glycation end products; RAS, rat sarcoma viral oncogene homolog; RCT, randomized controlled trial; RIPK1, receptor-interacting serine/threonine kinase 1; ROS, reactive oxygen species; RPS6, ribosomal protein S6; RSK, ribosomal S6 kinase; SAE, serious adverse event; SAH, subarachnoid hemorrhage; SDF-1α, stromal cell-derived factor 1 α; SHC, Src homology 2 domain-containing protein; SLC6A3, solute carrier family 6 member 3; SOD, superoxide dismutase; SOS, son of sevenless; SP1, specificity protein 1; STAT1, signal transducer and activator of transcription 1; STAT3, signal transducer and activator of transcription 3; STING, stimulator of interferon genes; TAK1, transforming growth factor-beta-activated kinase 1; TAO1/2, thousand-and-one amino acid protein kinase 1/2; TFR1, transferrin receptor 1; TLR4, Toll-like receptor 4; tMCAO, transient middle cerebral artery occlusion; TNF-α, tumor necrosis factor α; TPL2, tumor progression locus 2; TRAF6, tumor necrosis factor receptor-associated factor 6; TRIM45, tripartite motif-containing protein 45; Trx1, thioredoxin 1; UCP2, uncoupling protein 2; ULK1, unc-51-like autophagy activating kinase 1; USP14, ubiquitin-specific peptidase 14; VCAM-1, vascular cell adhesion molecule 1; VEGF-A, vascular endothelial growth factor A; ZNF460, zinc finger protein 460; ZO-1, zonula occludens 1.
