# Supplementary material for: Herbal-derived bioactive compounds in stroke recovery: from neuroprotective mechanisms to clinical evidence and translational challenges
Source: Front Neurol. 2026 Oct 16;17:1902978. doi: 10.3389/fneur.2026.1902978 (PMC13720355; doi:10.3389/fneur.2026.1902978)
Supplement: Supplementary file 1 [file Data_Sheet_1.pdf]

Table S1: Key Features of Hemorrhagic Stroke

Aspect	Details
Main Types	Intracerebral hemorrhage, Subarachnoid hemorrhage
Common Causes	Hypertension, aneurysm rupture, arteriovenous malformation (AVM)
Primary Mechanism	Vessel rupture → bleeding → increased intracranial pressure
Secondary Mechanisms	Hematoma formation, oxidative stress, inflammation, neuronal apoptosis
Clinical Complications	Edema, impaired perfusion, poor prognosis, high fatality rate
