# Supplementary material for: Herbal-derived bioactive compounds in stroke recovery: from neuroprotective mechanisms to clinical evidence and translational challenges
Source: Front Neurol. 2026 Oct 16;17:1902978. doi: 10.3389/fneur.2026.1902978 (PMC13720355; doi:10.3389/fneur.2026.1902978)
Supplement: Supplementary file 2 [file Image_1.pdf]

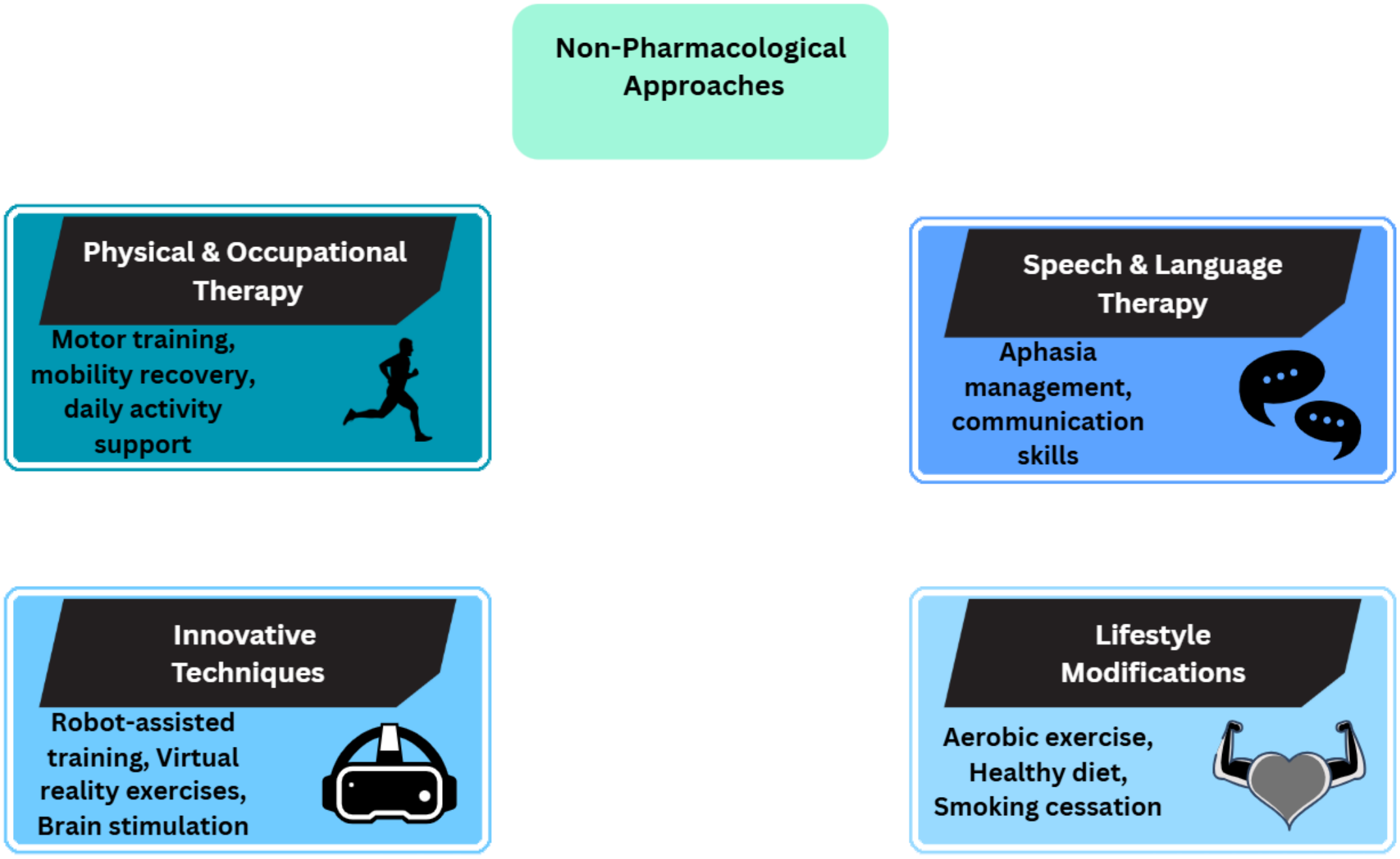

Fig. S1 | Non-Pharmacological Strategies in Stroke Rehabilitation

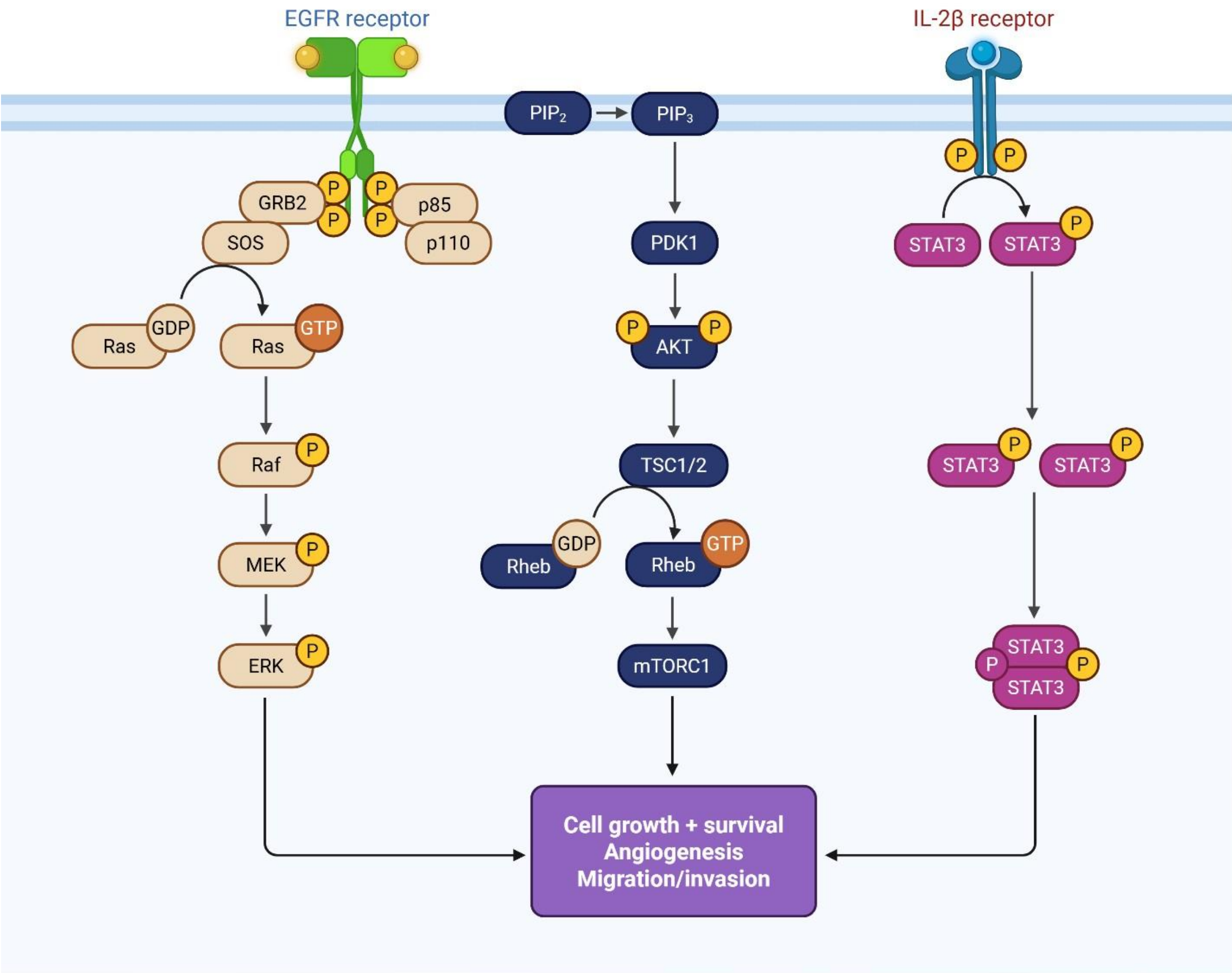

Fig. S2 | PI3K/Akt Signaling Pathway in Neuroprotection

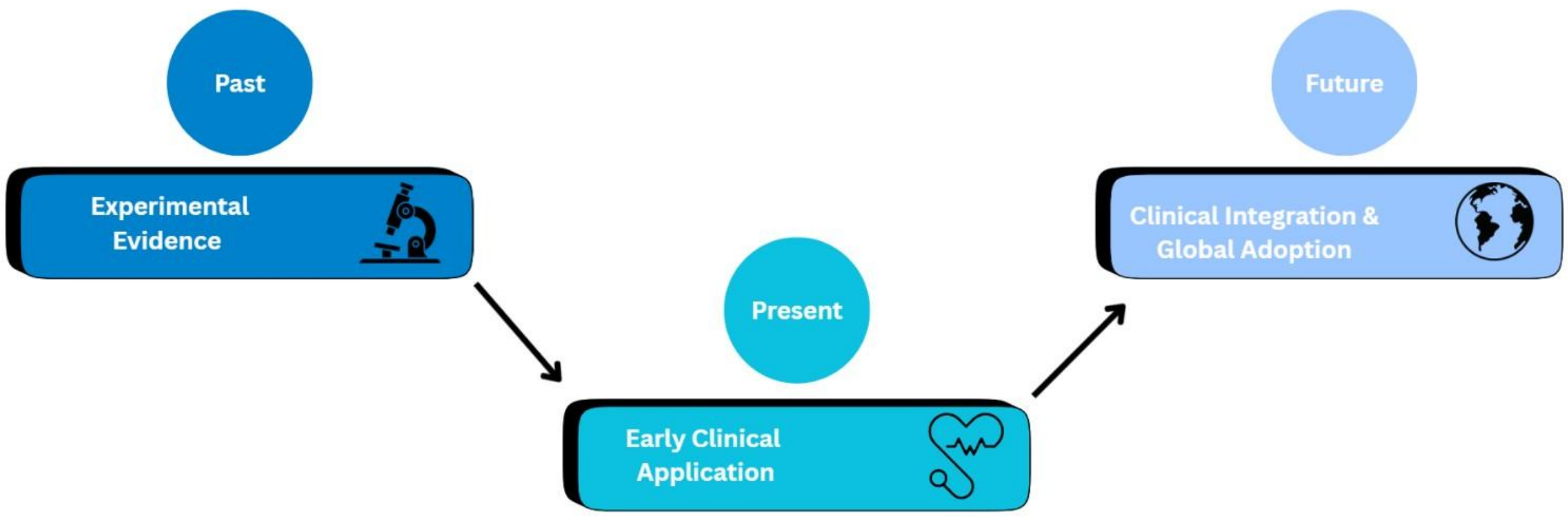

Fig. S3 | Translational Pathway of Herbal Bioactives from Research to Clinical Application in Stroke Rehabilitation
